# Sodium–Glucose Transporter 2 (SGLT2) Inhibitors and Iron Deficiency in Heart Failure and Chronic Kidney Disease: A Literature Review

**DOI:** 10.3390/life13122338

**Published:** 2023-12-13

**Authors:** Maria Tziastoudi, Georgios Pissas, Spyridon Golfinopoulos, Georgios Filippidis, Periklis Dousdampanis, Theodoros Eleftheriadis, Ioannis Stefanidis

**Affiliations:** Clinic of Nephrology, Faculty of Medicine, School of Health Sciences, University of Thessaly, 41334 Larisa, Greece; gpissas@msn.com (G.P.); spygolfin@yahoo.gr (S.G.); gfilippid@yahoo.gr (G.F.); dousdampanis@yahoo.gr (P.D.); teleftheriadis@yahoo.com (T.E.)

**Keywords:** heart failure, chronic kidney disease, anemia, iron deficiency, sodium–glucose co-transporter 2 inhibitors, SGLT2

## Abstract

Heart failure (HF) and chronic kidney disease (CKD) are associated with high mortality. In both disorders, impaired iron homeostasis, mostly in the form of a functional iron deficiency, is a frequent co-morbidity. In HF, functional iron deficiency and management by i.v. iron supplementation have been proven to affect both prognosis and functional capacity. In the same context, iron supplementation is routine for the adequate management of renal anemia in CKD. In numerous recent studies in HF and in CKD, sodium–glucose transporter 2 (SGLT2) inhibitor treatment has been proven to significantly reduce mortality. Furthermore, the same trials showed that these drugs alleviate iron deficiency and anemia. These effects of SGLT2 inhibitors may be due to an amelioration of inflammation with reduced interleukin-6 (IL-6) and to an enhancement of autophagy with increased sirtuin 1 (SIRT1), both associated with modified production of hepcidin and enhanced ferritinophagy. However, the exact pathogenic basis of the beneficial SGLT2 inhibitor action is not fully elucidated. Nevertheless, effects on iron homeostasis might be a potential explanatory mechanism for the powerful SGLT2 inhibitors’ cardiovascular and renal outcome benefits. In addition, the interaction between iron supplementation and SGLT2 inhibitors and its potential impact on prognosis remains to be clarified by future studies. This review represents a significant effort to explore the complex relationships involved, seeking to elucidate the intricate mechanisms by which SGLT2 inhibitors influence iron homeostasis.

## 1. Introduction

Heart failure (HF) is a very common disorder with an enhanced worldwide prevalence that parallels the aging of the general population [1,2] and is associated with considerably high morbidity and mortality [3]. It is defined by a wide-ranging spectrum of causative factors, which exhibit significant variability among different populations [4]. More specifically, in Africa, hypertensive heart disease is the main cause of HF [5], whereas in East Asia, due to lifestyle modifications, the primary cause of HF is ischemic heart disease [6].

Chronic kidney disease (CKD) is a chronic and progressive medical condition that affects a staggering number of the global population, exceeding 10%, which translates to more than 800 million people worldwide [7]. CKD exhibits distinct patterns of occurrence, with a higher frequency among the elderly, women, racial minority groups, and individuals dealing with comorbidities such as diabetes mellitus (DM) and hypertension [7]. Remarkably, CKD often coexists with HF, compounding the complexity of managing these conditions. Approximately 40% of patients with HF and a reduced ejection fraction (HFrEF) also have CKD, a fact that notably worsens their overall prognosis [8,9]. Conversely, around 30% of CKD patients suffer from HF [10]. This intricate interrelationship between CKD and HF results in a mutual amplification of severity, ultimately leading to a bad prognosis for affected individuals [11]. Therefore, there is an immediate need for an integrated approach to address these two disorders by acknowledging the intricate connections between them and the requirement for comprehensive care.

The interconnection between the cardiovascular (CV) and renal systems has long been acknowledged. In addition, iron deficiency and anemia are further highly prevalent co-morbidities in HF and CKD. Both of these disorders are associated with an unfavorable HF outcome: increased hospitalization and mortality as well as a worse quality of life [12]. The presence of CKD, with its potential to reduce erythropoietin production, plays a significant role in the pathogenesis of anemia in many cases of HF, exacerbating the overall health burden [13]. However, it is important to note that a substantial portion of CKD patients also suffer from iron deficiency anemia, which is primarily independent of deficient kidney erythropoietin production [14,15].

Among the available antidiabetic drug classes, sodium–glucose co-transporter 2 (SGLT2) inhibitors and glucagon-like peptide 1 receptor agonists (GLP-1RAs) have demonstrated remarkable efficacy in lowering the incidence of major adverse cardiovascular events, signifying their significant impact on the management of diabetes and its associated cardiovascular complications [16,17]. Moreover, it is noteworthy to highlight that SGLT2 inhibitors, in particular, have shown additional benefits beyond glycemic control. These agents have proven to be advantageous not only in diabetic individuals but also in nondiabetic patients with HF, underlining their potential as a valuable therapeutic option in the broader context of cardiovascular health [18].

In large clinical trials focused on individuals with type 2 diabetes mellitus (T2DM), SGLT2 inhibitors have been found to elevate hemoglobin and hematocrit levels, offering a potential remedy for anemia, a commonly encountered complication in patients with T2DM [19]. This beneficial effect of SGLT2 inhibitors appears to be consistent and independent of the primary diagnosis, whether it is CKD [20], HF [21], or DM [19]. Efficacy in alleviating anemia underscores the versatility of SGLT2 inhibitors as a potential therapy that transcends the boundaries of specific medical conditions. Furthermore, the reliability of these findings has been reinforced through various comprehensive meta-analyses, which have consistently affirmed the positive impact of SGLT2 inhibitors on hemoglobin and hematocrit levels [22,23].

The remarkable aspect of the alleviation of anemia following the administration of an SGLT2 inhibitor lies in its long-lasting effects and its apparent independence from preexisting anemia or even from an existing iron deficiency status. This is surprising taking into account that, for an adequate action of erythropoietin treatment, previous iron supplementation is necessary, especially in cases with a preexisting iron deficiency [24]. This surprising characteristic underscores the unique mechanisms of action associated with these inhibitors and raises important questions about how they influence erythropoiesis and red blood cell production.

Furthermore, the elevation of hematocrit and hemoglobin in these studies was closely related to the observed risk reduction in cardiovascular mortality and hospitalization for HF [25]. Specifically, a post hoc analysis of the EMPA-REG OUTCOME trial with diabetes type 2 patients unveiled that about 50% of the empagliflozin-induced clinical benefit is mediated by the elevation of hematocrit [26]. Clinical trials to address the clinical and prognostic significance of the SGLT2 inhibitor’s influence on anemia are currently being designed, providing a promising avenue for future research and potential breakthroughs in the fields of diabetes management and cardiovascular care [27].

The intriguing insights gleaned from these studies naturally give rise to several intriguing questions. First and foremost, there is a pressing need to delve into the mechanisms through which SGLT2 inhibitors influence iron homeostasis in patients dealing with HF and/or CKD. Understanding these underlying processes is pivotal in unraveling the full scope of the therapeutic potential of SGLT2 inhibitors in these specific patient populations. Additionally, a second essential question that arises pertains to how these effects on iron homeostasis might intersect with patient prognosis. Given that the positive, organ-specific influences of SGLT2 inhibitors are thought to be driven by pleiotropic effects, it becomes increasingly crucial to explore the intricate web of interactions between these agents and iron homeostasis within the context of HF and CKD. Last but not least, another challenging issue constitutes the interaction between SGLT2 inhibitors and iron supplementation treatment in HF or in CKD. This narrative review serves as a valuable endeavor in addressing these multifaceted relationships, aiming to shed light on the intricate mechanisms through which SGLT2 inhibitors exert their effects on iron homeostasis. By exploring these complex interrelationships, we strive to advance our understanding of the potential therapeutic benefits and mechanisms of SGLT2 inhibitors in the broader realm of cardiovascular and renal health.

## 2. Effects of SGLT2 Inhibitors

SGLT2 is a protein encoded by the solute carrier family 5 member 2 (*SLC5A2*) gene, located in the 16p11.2 region of the human genome. This transporter is primarily expressed in the kidney and is particularly prominent in the first segment (S1) of the proximal convoluted tubule (PT). SGLT2 plays a pivotal role as the major glucose transporter in the kidney, accounting for approximately 90% of glucose reabsorption by this vital organ. Tubular glucose re-absorption is sodium-dependent. SGLT2 transports two sodium ions (Na^+^) against one molecule of glucose. SGLT2 inhibitors, by blocking the co-transporter, inhibit the re-absorption of glucose and augment natriuresis. More sodium ions reach the thick ascending limb of the loop of Henle and the sodium sensor (NaK2Cl transporter) in the macula densa, leading to an activation of the tubuloglomerular feedback [28,29,30,31] with a reduction in glomerular capillary pressure, decrement of hyperfiltration, and albuminuria, and thus to an amelioration of glomerular and tubular injury [32,33,34,35]. Due to the associated glycosuria, SGLT2 inhibitors promote loss of weight and adipose tissue [36], which includes a reduction in epicardial fat with a cardioprotective influence [37].

In addition to their impact on glucose regulation and renal function, SGLT2 inhibitors exhibit a multifaceted array of effects, encompassing anti-inflammatory and anti-fibrotic and a substantial reduction in oxidative stress [38,39,40,41]. Notably, all these pleiotropic beneficial SGLT2 inhibitor effects [42] are independent of glycemic control in diabetes [34]. Many lines of evidence have emerged, shedding light on the diverse range of effects exerted by this drug class throughout the cardiovascular system, vascular system, and in the context of nonalcoholic fatty liver disease [43]. They also alter cardiac metabolism, as they increase the oxidation of fatty acids and ketogenesis, whereas they reduce the metabolism of carbohydrates [44]. This intricate interplay between SGLT2 inhibitors and various physiological processes underscores their capacity to influence not only diabetes and its associated complications but also the broader spectrum of cardiovascular health and metabolic pathways.

SGLT2 inhibitors also improve oxygen delivery. This is achieved through the stimulation of renal erythropoietin (EPO) secretion, a hormone responsible for regulating red blood cell production. As a result, SGLT2 inhibitors promote the formation of new red blood cells, leading to an increase in hematocrit levels, which, in turn, holds the potential to significantly improve cardiac function [25,45]. These remarkable effects on oxygen delivery and blood composition have sparked discussions about the potential applicability of SGLT2 inhibitors in patients with HF with preserved ejection fraction (HFpHF) [46].

## 3. Iron Metabolism

Iron exists mainly in two forms within the human body: the trivalent iron ion (Fe^2+^) and the ferrous ion (Fe^2+^). The body absorbs and utilizes iron primarily in the Fe^2+^ form, while it transports it in the Fe^2+^ form [47]. The uptake of Fe^2+^ occurs through the divalent metal transporter 1 (DMT1), which is located on the luminal side of small intestinal epithelial cells [48]. Following absorption in the small intestine, a portion of Fe^2+^ is utilized to synthesize ferritin in intestinal mucosal epithelial cells, while the remainder enters the bloodstream [49].

Ferritin facilitates the transfer of Fe^2+^ to blood circulation through ferroportin (FPN), the sole known cellular iron-exporting protein, situated on the basolateral membrane side. The regulation of serum iron levels is tightly controlled by the interaction between hepcidin and FPN [50]. In instances of in vivo iron deficiency, decreased hepcidin expression allows iron to be released into the plasma via FPN [51].

Upon entering the bloodstream, Fe^2+^ undergoes conversion into Fe^2+^ facilitated by hephaestin (HP) or ceruloplasmin. The resulting Fe^2+^ then binds with transferrin, the primary iron transporter, and is transported to various tissues [52]. Transferrin attaches to transferrin receptor 1 (TFR1) and transferrin receptor 2 (TFR2) on the cell surface of iron-deficient cells, entering the cells in a controlled manner [53].

The cornerstone of diagnosing iron deficiency primarily relies on the assessment of serum ferritin levels, which serve as a valuable indicator of cellular iron stores within the body. In healthy individuals, iron deficiency is diagnosed at a serum ferritin level < 30 μg/L. However, in the context of HF and CKD, the diagnostic criteria for iron deficiency are more stringent, with a ferritin level of less than 100 μg/L or a transferrin saturation (TSAT) of 20% or less when serum ferritin is below 300 μg/L [12]. These laboratory findings are indirect markers, reflecting in a clinically relevant manner, cytosolic bioreactive iron (Fe^2+^). Other biomarkers, except for ferritin and iron levels as well as transferrin saturation, include unsaturated iron-binding capacity, soluble transferrin receptor, plasma hepcidin, and erythropoietin [21]. Certainly, iron deficiency, whether absolute or relative (functional), occurs when the cytosolic iron level is insufficient to support the requirements of heme biosynthesis for proper erythropoiesis. The diagnostic criteria in HF and CKD reflect the increased complexity and multifactorial nature of iron deficiency in these patient populations, underlining the importance of precise assessment and tailored management to address their unique iron-related challenges.

## 4. Iron Deficiency Anemia in Heart Failure and Chronic Kidney Disease

Iron deficiency represents a state characterized by a diminished delivery of iron to cells, resulting in a cascade of biological repercussions. This deficiency ultimately leads to impaired production of essential components, such as heme and iron–sulfur compounds, which play pivotal roles in the biosynthesis of hemoglobin and oxidative phosphorylation, a critical cellular energy-producing process. This impairment leads thus to reduced erythropoiesis by erythroid precursors in the bone marrow and to reduced cellular production of ATP in mitochondria (e.g., the mitochondria of cardiomyocytes). Therefore, iron deficiency exerts a wide-ranging influence on cellular and systemic functions, highlighting its crucial role in maintaining overall health and well-being.

Iron deficiency is a complex condition that can manifest itself in various forms, including both absolute and relative (functional) iron deficiency. Absolute iron deficiency represents a situation where there is either a complete absence or a severe reduction in the total body iron stores. Iron stores are typically found in macrophages and hepatocytes, and their scarcity can have profound effects on various physiological processes. On the other hand, functional iron deficiency refers to a condition in which the body possesses an adequate amount of iron stores, but it struggles to mobilize these stores effectively for critical functions like erythropoiesis (red blood cell production) or other cellular processes. In functional iron deficiency, the issue is not the lack of iron stores but rather an impairment in the ability to utilize the available iron stores efficiently.

The presence of absolute iron deficiency in HF and individuals with CKD often stems from a combination of factors. These factors encompass reduced iron intake, such as anorexia, which can limit the body’s access to this essential mineral. Additionally, impaired iron absorption, potentially caused by gastrointestinal edema, can further hinder the body’s ability to maintain adequate iron stores. Moreover, the use of anticoagulation and anti-platelet aggregation drugs in these patient populations can increase the risk of gastrointestinal blood loss, contributing to the depletion of iron stores [12]. In the context of CKD, another notable contributor to absolute iron deficiency is iatrogenic blood loss. This can occur through frequent blood tests that are necessary for monitoring the condition, as well as losses that may happen in the vascular access and extracorporeal circuits commonly used in the management of CKD patients [15]. These factors compound the challenge of maintaining sufficient iron stores in individuals with CKD, necessitating careful management and monitoring of iron levels to ensure optimal health outcomes.

**Figure 1:**
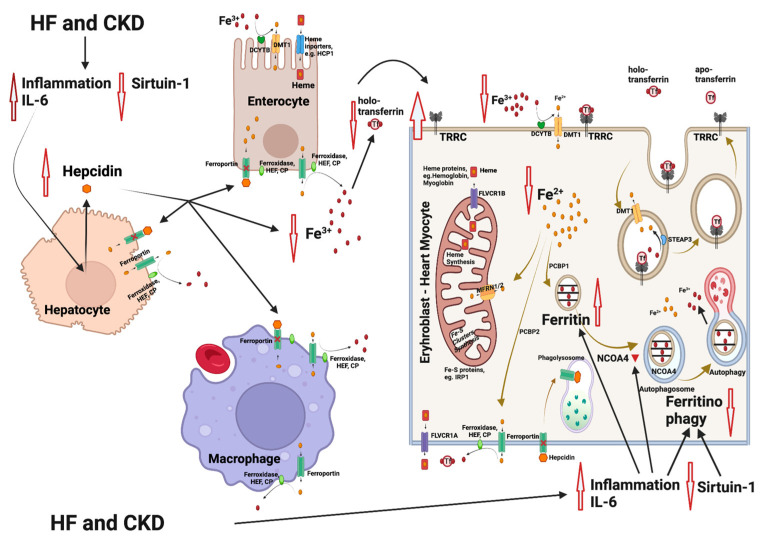
Mechanisms involved in iron deficiency and iron deficiency anemia in patients with heart failure (HF) and chronic kidney disease (CKD). HF and CKD lead to activation of inflammation through elevation of interleukin-6 (IL-6) levels and to an inhibition of sirtuin 1 (SIRT1) expression. As a result, hepcidin is upregulated, leading to an inhibition of the Fe^2+^ efflux from enterocytes and macrophages (resident in spleen and liver). The Fe^2+^ flow to the extracellular space and to the blood is blocked, followed by a reduction in the intracellular Fe^2+^ in heme and/or Fe-S-cluster protein-producing cells (e.g., erythroblasts and cardiomyocytes). Parallel, IL-6 also inhibits the nuclear receptor coactivator 4 (NCOA4), which normally mediates ferritinophagy, leading to the release and export of iron to the cytosol for use in various processes (e.g., mitochondrial heme synthesis). NCOA4 inhibition leads to enhanced entrapment of iron in ferritin and to a further reduction in the available cytoplasmic Fe^2+^. The resulting state is functional iron deficiency [55,56]. Abbreviations: CP: ceruloplasmin, DMT1: divalent metal transporter 1, DCYTB: duodenal cytochrome b, FLVCR1A/B: feline leukemia virus subgroup C receptor 1A/B, HEF: hephestin, IRP1: iron-regulating protein 1, MFRN1/2: mitoferrin 1/2, NCOA4: nuclear receptor coactivator 4, PCBP1/2: poly r(C)-binding protein 1/2, SGLT2i: SGLT2 inhibitors, SIRT1: sirtuin 1, STEAP: six-transmembrane epithelial antigen of prostate, Tf: transferrin, TFRC: transferrin receptor. Created with BioRender.com (accessed on 10 November 2023).

Relative iron deficiency in HF often arises from a state of chronic inflammation, which is characterized by elevated levels of inflammatory cytokines, resembling the pattern seen in anemia from chronic disease (Figure 1). This inflammatory milieu includes an increase in cytokines, notably interleukin-6 (IL-6), which exerts a cascade of effects on iron metabolism. IL-6 and other inflammatory mediators can upregulate the production of hepcidin in the liver. Hepcidin, in turn, plays a central role in regulating iron balance by inactivating ferroportin, the primary cellular iron exporter. This results in an inhibition of iron mobilization from its cellular stores, such as hepatocytes and macrophages. Importantly, this hepcidin-mediated inactivation of ferroportin in enterocytes within the gastrointestinal tract further hampers iron absorption, contributing significantly to the development of iron deficiency [54]. There is a significant reduction in iron and transferrin saturation in the blood and an upregulation of transferrin receptor (TfR) on the cell membranes of heme-synthesizing cells. Apart from the effects on hepcidin, the inflammatory mediators promote ferritin synthesis, resulting in the entrapment of iron in its intracellular stores, independent of iron concentration adequacy [12].

In the context of CKD, the state of chronic inflammation is a recurrent theme, closely linked to enhanced hepcidin levels and contributing to the development of functional iron deficiency. In humans, individuals with chronic infections or severe inflammatory diseases exhibit elevated levels of urinary hepcidin. In hepatic cell cultures, the expression of hepcidin can be stimulated by cytokines, especially interleukin-6 (IL-6) [57]. An additional reason for increased hepcidin in CKD is reduced renal clearance [58,59]. More specifically, in chronic kidney disease, elevated plasma hepcidin levels, driven by inflammation and compromised renal clearance, impede duodenal iron absorption and result in the sequestration of iron in macrophages [60,61]. Erythropoietin deficiency is the main cause of anemia in CKD, and, according to guidelines, an erythropoiesis-stimulating agent (ESA) treatment is standard [62]. ESA administration in CKD may be an additional cause of a functional iron deficiency, which results from the enhanced iron need for ESA-induced erythropoiesis [15].

Treatment of anemia with erythropoietin in HF has no impact on the overall prognosis, and darbepoietin has even been associated with an increased rate of thromboembolism [12]. In CKD, target hemoglobin levels are 10–11.5 g/dL, while a hemoglobin level > 13 mg/dL is associated with increased cardiovascular risk and is contraindicated [63]. Intravenous iron supplementation in iron-deficient patients with HF had a positive impact on morbidity (hospitalization) and on cardiovascular and overall mortality [64,65]. In a CKD population study, the prevalence of anemia was 20.6% [66]. In this large observational trial, iron deficiency anemia, either absolute (TSAT ≤ 20%, ferritin < 100 μg/L) or relative (functional TSAT ≤ 20%, ferritin 100–500 μg/L), was associated with an increased cardiovascular hospitalization rate. Specifically, in the group with functional iron deficiency anemia (TSAT ≤ 20%, ferritin 100–500 μg/L) and in the group with ferritin levels > 500 μg/L, a higher risk of mortality was observed [66]. However, randomized clinical trials concerning iron supplementation in CKD are missing [67].

A noninferiority trial involving 2141 patients focused on the maintenance of intravenous iron was conducted exclusively in chronic hemodialysis cases. Patients were randomly assigned to receive high-dose iv iron sucrose proactively (400 mg monthly, independent of ferritin level < 700 μg/L or TSAT < 40%) or low-dose iron sucrose reactively (given if ferritin < 200 μg/L or TSAT < 20%). At the follow-up of 2.1 years, there was no difference between the groups in the composite end-point (nonfatal myocardial infarction, nonfatal stroke, hospitalization for HF, or death) [67].

## 5. Influence of SGLT2 Inhibitors on Erythropoiesis and Iron Homeostasis

It is known that SGLT2 inhibitors increase both hemoglobin levels and hematocrit in T2DM patients with relatively normal renal function [45,68]. These findings were replicated in the context of the CREDENCE and DAPA-HF trials, which included patients with CKD and HF, respectively, and these effects were consistent and long-sustained [20,21]. Although, from the recent literature, it can be extrapolated that the ancillary effect of SGLT2 inhibitors present in DM2 patients with HF and CKD could delay the onset of anemia in cardiorenal syndrome, to date, it is not considered a treatment for nephrogenic anemia [69]. Many investigators have attributed this rise to the contraction of plasma volume. Such a contraction effect, however, would be transitory and not a long-sustained elevation of hematocrit, as seen in certain clinical trials [70]. According to another hypothesis, which was formulated relatively early and is still in discussion, it is postulated that SGLT2 inhibitors enhance erythropoietin production by having an indirect influence on the peritubular interstitial fibroblasts [70]. SGLT2 inhibition reduces glucose reabsorption and metabolic stress in proximal tubule epithelial cells (PTECs), leading to a reversal of interstitial hypoxia, fibrosis, and cellular damage. Peritubular interstitial fibroblasts regain viability and their facility to produce erythropoietin (O’Neill et al., 2015) [71].

In addition, SGLT2 inhibitors have a direct effect on hypoxia-inducible factors (HIFs) [72], inhibiting HIF-1α and activating HIF-2α. The inhibition of HIF-1α may underlay the cardiovascular benefits of the SGLT2 inhibitors [72]. HIF-2α is the primary stimulus for erythropoietin production [73]. Enhanced erythropoietin levels were noted up to 12 weeks after initiation of the SGLT2 inhibitors in several placebo-controlled trials in HF and T2DM [68,74,75]. The activation of HIF-2α is probably mediated by the upregulation of the starvation factor sirtuin 1 (SIRT1), a known enzymatic modulator of HIF [76]. It is worth mentioning that daprodustat, a HIF propyle hydroxylase that corrects anemia, when combined with dapagliflozin in three patients with acute myeloid leukemia, was well tolerated [77].

An erythropoietin rise alone cannot lead to an increase in red cell production without an adequate intracellular iron concentration. SGLT2i, however, increases hematocrit independent of iron adequacy. In this context, SGLT2 inhibitors have been shown to interfere directly with iron homeostasis, leading to a decrease in hepcidin, ferritin, and transferrin saturation and a rise in soluble transferrin receptors (sTfRs) in clinical trials [21,75]. These findings were confirmed in placebo control studies in patients with T2DM or HF, including proteomics studies [78] and mRNA expression analysis [79].

SGLT2 inhibitor treatment limits the inflammatory state and simultaneously promotes nutrient deprivation signaling, especially the expression of SIRT1 [80]. Reduced inflammation leads to a reduced production of hepcidin and ferritin. Furthermore, SIRT1 directly stimulates HIF2α signaling and ferritin catabolism. The final result of the above effects is an enhancement of the available iron in both the blood and cytoplasm.

The rise of cytosolic bioreactive iron, together with the rise of erythropoietin induced by SGLT2i, facilitates erythropoiesis in the bone marrow. Cytosolic iron further facilitates mitochondrial ATP production in cardiomyocytes. Enhanced ATP production in the failing myocardium might be the basis of the beneficial effects of SGLT2 inhibitors in HF. A similar mechanism might be attributed to the outcome benefit after intravenous iron supplementation in iron-deficient HF patients. These parallel actions of SGLT2 inhibitors on bone marrow and myocardium might be the reason why the elevation of hemoglobin is one main predictor of cardiovascular prognosis in clinical trials.

It also aught to be noted that SGLT2 inhibitors exert their benefit on red blood cell production even in patients who are iron-deficient before treatment. This observation clearly indicates, first, that SGLT2 inhibitor treatment directly reverses the effects of iron deficiency without any need for iron supplementation, and second, that in most cases with HF, iron deficiency is rather functional than absolute.

**Figure 2:**
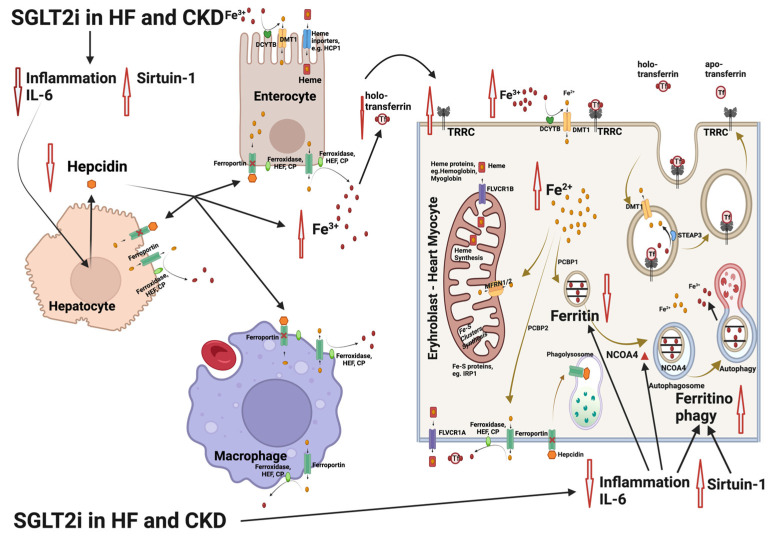
Interference of the sodium glycose co-transporter 2 (SGLT2) inhibitor treatment with the mechanisms involved in iron deficiency and iron deficiency anemia in patients with heart failure (HF) and chronic kidney disease (CKD). SGLT2 inhibitors limit inflammation, lowering interleukin-6 (IL-6) expression and upregulating sirtuin 1 (SIRT1). The result is an inhibition of hepcidin production, leading to increase in Fe^3+^ both in plasma and inside the cells. In parallel, nuclear receptor coactivator 4 (NCOA4) is enhanced as IL-6 and inflammation is limited. NCOA4 mediates ferritinophagy, leading to release of iron from ferritin entrapment and enhancing the available cytoplasmic Fe^2+^ for use in various processes (e.g., mitochondrial heme synthesis). The resulting state is an amelioration of functional iron deficiency, which is commonly observed in patients with HF and CKD [55,56]. Abbreviations: CP: ceruloplasmin, DMT1: divalent metal transporter 1, DCYTB: duodenal cytochrome b, FLVCR1A/B: feline leukemia virus subgroup C receptor 1A/B, HEF: hephestin, IRP1: iron-regulating protein 1, MFRN1/2: mitoferrin 1/2, NCOA4: nuclear receptor coactivator 4, PCBP1/2: poly r(C)-binding protein 1/2, SGLT2i: SGLT2 inhibitors, SIRT1: sirtuin 1, STEAP: six-transmembrane epithelial antigen of prostate, Tf: transferrin, TFRC: transferrin receptor. Created with BioRender.com (accessed on 10 November 2023).

Furthermore, treatment with SGLT2 inhibitors leads invariably to a decrease in serum ferritin and transferrin saturation in laboratory findings that meet the typical current diagnostic criteria for an ensuing iron deficiency (Figure 2). After 12 months in the DAPA-HF trial, patients on dapagliflozin vs. placebo developed iron deficiency, according to the current diagnostic criteria, far more frequently (70%). However, dapagliflozin continued to induce uninterrupted, significant erythropoiesis and a rise in hemoglobin (Docherty et al., 2022) [21]. These intriguing findings raise the logical question about the clinical reliability of the laboratory diagnostic markers currently in use for iron deficiency, especially when patients are receiving SGLT2i.

In patients with HF under treatment with SGLT2 inhibitors, intravenous iron supplementation is probably unnecessary, even if they meet the criteria for iron deficiency. It would even induce an excessive rise in cytosolic iron levels with negative effects, leading to excessive phospholipid oxidation and to ferroptosis, a special form of programmed cell death [80]. Importantly, SIRT1 over-expression induced by SGLT2 inhibitor treatment can suppress ferroptosis and prevent these deleterious consequences of increased cytosolic iron [81,82]. SIRT1 has recently been shown to control ferroptosis by influencing various target genes, among which is *p53*, the deacetylation of which suppresses ferroptosis [83]. Indeed, many lines of evidence indicate the crucial role of SIRT1 in the regulation of ferroptosis. More specifically, nephrolithiasis deacetylation of p53 by SIRT1 suppressed ferroptosis, alleviating calcium–oxalate (CaOx) crystal-induced damage [84,85]. SIRT1 inhibits ferroptosis-induced myocardial cell death through the p53/SLC7A11 axis [82]. Another target of SIRT1 is nuclear factor erythroid2-related factor 2 (NRF2), which prevents lipid peroxidation, another key contributor to ferroptosis. SIRT1 can manifest anti-ferroptotic effects through the activation of NRF2, a key regulator in the antioxidant stress response, which also affects iron metabolism as it enhances ferritin formation and induces ferroportin [83]. Together, these discoveries demonstrate that NRF2 can efficiently suppress ferroptosis. The inhibitory impact of SIRT1–NRF2 on ferroptosis has been confirmed across various diseases, such as doxorubicin-induced cardiomyopathy [82], diabetic retinopathy [86], liver injury triggered by acetaminophen overdose [87], and depression and anxiety-like behavior [88], and it promotes neurocognitive recovery [89]. Last but not least, SGLT2 inhibitors and intravenous iron supplementation both have, as a single treatment, a positive impact on HF; however, the effects of their combined use have not yet been tested in any clinical trial. Therefore, their co-administration in HF treatment is currently not proposed [90].

## 6. Conclusions

Recent studies have shown that SGLT2 inhibitors are effective in reducing mortality and in improving iron deficiency and anemia in individuals with HF and CKD. The findings suggest that the impact of SGLT2 inhibitors on iron homeostasis might explain the significant cardiovascular and renal benefits observed in these studies. While studies implicate a link between SGLT2 inhibitors and the modulation of hepcidin and sirtuin 1, the exact pathogenic basis of this relationship has not been fully clarified. Furthermore, the interaction between SGLT2 inhibitors and iron supplementation treatment in HF or in CKD remains an interesting question for future research. It is important to determine in what way the combination of SGLT2 inhibitors and iron supplementation influences prognosis.
